# *Nigella sativa* Oil Mouth Rinse Improves Chemotherapy-Induced Oral Mucositis in Patients with Acute Myeloid Leukemia

**DOI:** 10.1155/2019/3619357

**Published:** 2019-10-30

**Authors:** Saad Abdulrahman Hussain, Hazha Abdulah Mohammed Ameen, Mohammed Omer Mohammed, Khadija Muhamed Ahmed, Rebaz Hama-Gareb Ali, Banaz Mubarak Safar, Kamal Ahmed Saeed

**Affiliations:** ^1^Department of Pharmacology and Toxicology, Faculty of Pharmacy, Al-Rafidain University College, Baghdad, Iraq; ^2^Department of Medicine, College of Medicine, University of Sulaimani, Kurdistan Region, Sulaimani, Iraq; ^3^Department of Oral Pathology, College of Dentistry, University of Sulaimani, Kurdistan Region, Sulaimani, Iraq; ^4^Department of Pharmaceutics, College of Pharmacy, University of Sulaimani, Kurdistan Region, Sulaimani, Iraq; ^5^Hiwa Oncology Hospital, Sulaimani, Kurdistan Region, Iraq; ^6^Department of Surgery, College of Medicine, University of Sulaimani, Kurdistan Region, Sulaimani, Iraq

## Abstract

**Objective:**

The present study aims at evaluating the beneficial effect of *Nigella sativa* (NS) oil mouth rinse in the management of chemotherapy- (CT-) induced oral mucositis (OM) in patients with acute myeloid leukemia (AML).

**Methods:**

Fifty-four AML patients were participated in this study and randomly allocated to either the test group or a control group. The patients of the test group received NS oil mouth rinse during 28-day CT, while the participants of the control group received a “magic mouthwash” formula. The primary outcome of this study was the incidence and severity of CT-induced OM in terms of erythema and ulcer. The secondary outcomes were the pain severity score, swallowing function, and the salivary concentrations of interleukin-6 (IL-6) and tumor necrosis factor-alpha (TNF-*α*).

**Results:**

NS oil mouth rinse attenuated the progression of CT-induced OM compared with the control formula (AUC = 5.9 vs. 38.4, *P* < 0.05) and significantly decreased the erythema and ulceration scores (AUC of total OMAS = 11.4 vs. 85.9, *P* < 0.001) compared with the magic mouthwash formula. It also reduced the pain score and enabled all the participants of this group to consume normal food during treatment. It significantly decreased salivary IL-6 (AUC = 7376 vs. 16599, *P* < 0.001), while the changes of TNF-*α* levels were not significant (AUC = 676.9 vs. 885.2, *P* > 0.05).

**Conclusions:**

NS oil mouth rinse is effective in attenuating the severity of CT-induced OM and improves the pain and swallowing function in AML patients.

## 1. Introduction

The use of systemic chemotherapy (CT) is the first-line treatment of acute myeloid leukemia (AML) [1]. However, it can induce many side effects including oral mucositis (OM) [2, 3]. Oral mucositis is one of the most commonly recognized disabling complications of both chemotherapy and radiotherapy [4] and mostly attributed to complex pathophysiology [5]. This drug-induced adverse reaction was reported in 20–40% of cancer patients receiving CT and in all patients treated with head and neck radiotherapy [6]. There are many treatment-related problems caused by OM for both the patients and the healthcare system including dysphagia [7], debilitating pain [8], and elevated patients' care costs [9]. Moreover, high incidence of infections [10], dose reduction and treatment discontinuation [6], and frequent hospitalization [11] were associated with the impaired quality of life of leukemia patients [12]. Many approaches have been followed to prevent and/or treat CT-induced OM, including oral care protocols and the use of drug combination that target various causes and consequences of OM [13]. The available mainstay approaches for prevention/treatment of OM include the use of laser therapy or cryotherapy during chemotherapy, use of amifostine (a cytoprotectant) [14], chlorhexidine mouthwash, and Chinese medicine [15], in addition to the use of a colony-stimulating factor [16]. Currently, at least in our center and most of the hematology units, a mouth rinse containing sodium bicarbonate and normal saline is used as a preventive measure. Meanwhile, mouthwash formulas containing diphenhydramine, nystatin, lidocaine, and chlorhexidine (magic mouthwash) are used as a treatment after the appearance of the OM symptoms. However, this multidrug cocktail may increase the incidence of side effects and may not completely satisfy the patients [17]. Natural products have been tried in experimental and clinical settings a few decades ago to treat CT-induced OM because of their appropriate efficacy and limited adverse effects [18]. *Nigella sativa* (NS), also known as black cumin, belongs to the Ranunculaceae family and has been safely utilized orally and topically as an alternative medicine since ancient times [19]. The volatile oil obtained from the seeds contains many constituents such as thymoquinone, carvacrol, thymol, *α*- and *β*-pinene, 4-terpineol, and nigellone, among others [20], and the therapeutic benefit of the seed extract is well characterized [21]. In this regard, *Nigella sativa* (NS) oil is tried in different animal models of radiation-induced mucositis because of its pleiotropic activities [22, 23] with promising pieces of evidence of successful outcomes. Accordingly, the present study aims at evaluating the effects of *Nigella sativa* oil as a mouth rinse against CT-induced OM in patients with acute myeloid leukemia.

## 2. Materials and Methods

### 2.1. Study Design and Participants

The current randomized, open-label controlled study with two parallel arms was performed at Hiwa Oncology Hospital, Sulaimani City, Iraq, from May 2017 to June 2018. The study protocol was approved by the Research Ethics Committee of the College of Medicine, University of Sulaimani (REC-45-13/2/2017), and the local Medical Ethics Committee of Hiwa Oncology Hospital. Written informed consent was obtained from each patient before enrollment and after providing all the required details on the nature of the study according to the principles of the Declaration of Helsinki. The AML patients who were assigned as candidates to receive the routinely followed “3 + 7” chemotherapy protocol (3 days of 60–90 mg/m^2^ daunorubicin and 7 days of 100–200 mg/m^2^ cytarabine) were selected as eligible samples. The inclusion criteria were patients diagnosed with AML, age > 18 years, prepared to receive CT for the first time, no evident signs of mucositis before the initiation of CT, lack of underlying serious diseases, and lack of compromised immune functions. The exclusion criteria were previous CT, presence of other malignancy within the last 5 years, presence of serious infection, and the presence of any oral ulcers before starting CT.

### 2.2. Recruitment and Randomization

**Figure 1:**
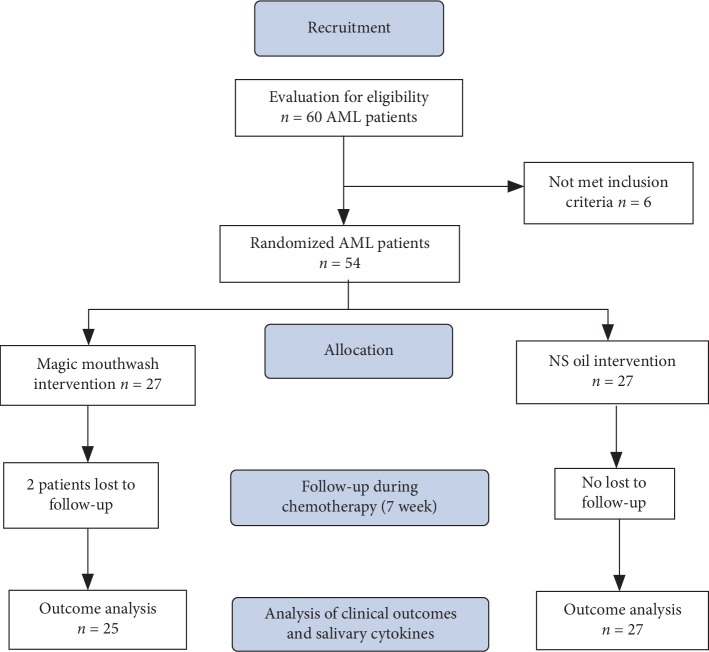
Flowchart of the study.

A total of 60 AML patients were screened for eligibility; 6 patients were not eligible and 54 patients, who provide the signed written informed consent, were enrolled and randomized to one of two groups (magic mouthwash arm that includes 27 patients, utilized as control; the NS oil arm that includes 27 patients) using simple randomization and an allocation ratio of 1 : 1. Only 52 patients completed the study (Figure 1). The allocation sequence and preparation of the magic mouthwash formula and the NS oil mouth rinse are performed by subjects not involved in the recruitment, data collection, and analysis. The NS oil formula was commercially available from a well-recognized supplier (BARRY Int. PVT., LTD, Karachi, Pakistan). The magic mouthwash formula was freshly prepared daily before use and contains nystatin 100,000 U, tetracycline 0.02%, lidocaine 0.5%, and dexamethasone 0.5%. The patients in both groups received the NS oil mouth rinse and the magic mouthwash topically as a mouth rinse (10 ml each 6 hr) daily, starting from the first day after the initiation of CT up to day 28 (the end of the CT).

### 2.3. Outcome Measurement and Data Collection

#### 2.3.1. Oral Mucositis Grading Scales

To control the confounding variables, the enrolled patients were carefully evaluated before starting the study and those with oral cavity disorders were excluded [24]. Furthermore, considering that the type of CT followed is known to cause oral mucositis, all the AML patients were receiving the “3 + 7” induction CT protocol. The primary outcome of the study was the grading and evaluation of OM, assessed at five time intervals: baseline, day 4, day 12, day 18, and day 28 after initiation of CT. The WHO and the National Cancer Institute (NCI) mucositis grading scales were utilized to assess the OM severity. In both scales, four grades of OM are included (stage 0 = lack of ulcers, stage 1 = pain and erythema, stage 2 = erythema and ulcer, stage 3 = ulcer and large erythema and the patient cannot eat solid food, and stage 4 = mucositis is in severe stage and oral feeding is not possible) [25].

#### 2.3.2. Assessment of Oral Mucositis

Additionally, the Oral Mucositis Assessment Scale (OMAS), a newly validated scale that measures the objective and subjective findings independently, was also tried for OM assessment. Briefly, OMAS assesses nine oral sites for erythema and ulceration: the upper lip, the lower lip, the right and left cheek, the right and left lateral tongue, the ventral surface of the tongue, the floor of the mouth, and the soft palate. The assessment of ulceration in each site was scored from 0 to 3, with 0 representing no clinical lesion, 1 representing an ulcer <1 cm^2^, 2 representing an ulcer of 1–3 cm^2^, and 3 representing an ulcer >3 cm^2^. The erythema was scored between 0 and 2, with 0 representing no erythema, 1 representing nonsevere erythema, and 2 representing severe erythema. The total ulcer score ranged between 0 and 27, and the total erythema score ranged between 0 and 18. The sum of the scoring for the oral cavity provided an overall score that reflects the mucosal damage [26].

#### 2.3.3. Assessment of Pain

The patients' reported outcomes were assessed by 2 visual analog scales (VAS) for pain and swallowing on a vertical line. Patients indicated their pain perception and ability to function depending on the eating ability of normal, soft, liquid, and no food or liquid food [27].

#### 2.3.4. Assessment of Salivary IL-6 and TNF-*α*

The secondary outcome of the study was the evaluation of salivary concentrations of IL-6 and TNF-*α*. Briefly, a minimum of 2 ml of unstimulated saliva was collected after oral rinse with water at room temperature. The saliva samples were collected at least three hours after the last meal, and the patients were asked to avoid drinking one hour before sampling. Due to the circadian rhythm nature of salivation, samples were collected from 9 to 11 am and immediately frozen at −40°C. Time points of the collection were the same as that of the clinical assessment (D0, D4, D12, D18, and D28). Based on sandwich ELISA technique, the salivary contents of IL-6 and TNF-*α* were evaluated using a commercial ready-made ELISA kit (Elabscience Biotechnology Inc., Texas, USA).

### 2.4. Statistical Analysis

The data are presented as numbers and percentages for categorical variables and as mean ± SEM or mean ± SD for continuous variables when applicable. Data analyses were performed utilizing SPSS version 21 and GraphPad Prism 6.1 software. Unpaired Student's *t*-test and ANOVA supported by Bonferroni's *post hoc* analysis were used for the analysis of continuous variables. Meanwhile, the chi-square test, Fisher's exact test, Mann–Whitney test, and Kruskal–Wallis supported by Dunn's multiple comparison *post hoc* tests were used for the analysis of the categorical data. *P* values <0.05 were considered statistically significant.

## 3. Results

### 3.1. Patients' Characteristics

Figure 1 shows that, of the 27 AML patients in each arm of the study, 2 participants died during the first week of CT treatment. In Table 1, the data collected from the enrolled AML patients in both groups were statistically analyzed and found comparable in terms of the demographic and disease-related characteristics as the comparison of some primary features at baseline of the two groups revealed no statistical differences between them (Table 1).

### 3.2. Primary Outcome: Clinical Scores

**Figure 2:**
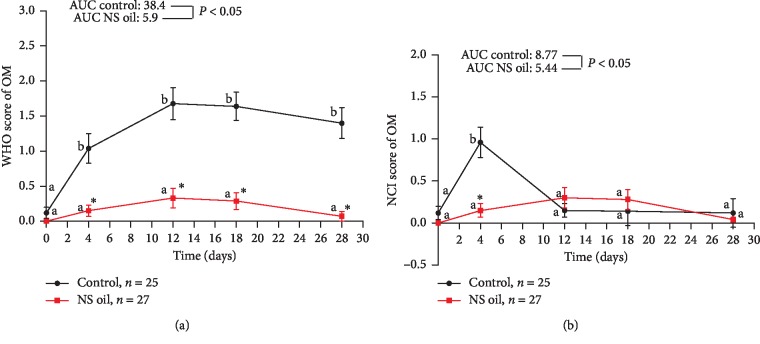
Effect of *Nigella sativa* oil (NS) mouth rinse on the (a) WHO and (b) NCI scales of chemotherapy-induced OM in AML patients. Values are presented as mean ± SD; values with nonidentical letters (a, b) within the same group are significantly different (*P* < 0.05); ^*∗*^significantly different compared with the control at the same time point (*P* < 0.05).

**Figure 3:**
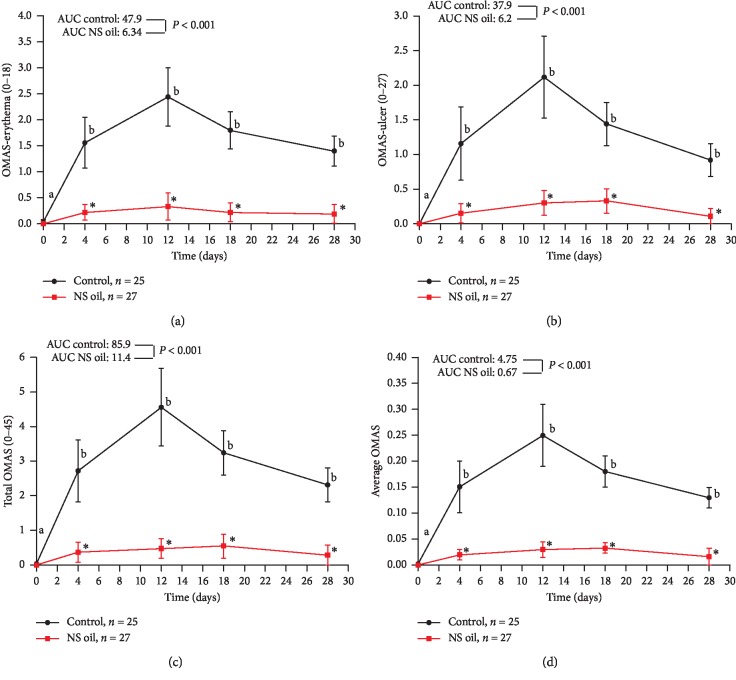
Effect of *Nigella sativa* oil (NS) mouth rinse on the (a) OMAS-E, (b) OMAS-U, (c) total OMAS, and (d) average OMAS of chemotherapy-induced OM in AML patients. Values are presented as mean ± SD; values with nonidentical letters (a, b) within the same group are significantly different (*P* < 0.05); ^*∗*^significantly different compared with the control at the same time point (*P* < 0.05).

**Figure 4:**
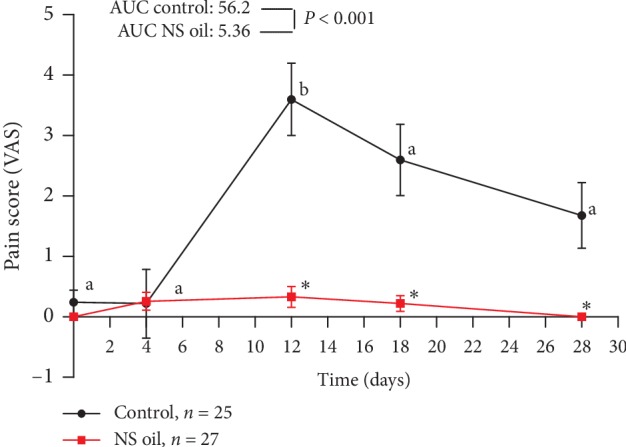
Effect of *Nigella sativa* oil (NS) mouth rinse on the pain score of chemotherapy-induced OM in AML patients. Values are presented as mean ± SEM; values with nonidentical letters (a, b) within the same group are significantly different (*P* < 0.05); ^*∗*^significantly different compared with the control at the same time point (*P* < 0.05).

Evaluation of OM severity using the MOH scale in the study groups showed the absence of predictable signs of OM at baseline, and no significant difference between them was reported at that time (Figure 2(a)). The mean score of severity was significantly increased in the magic wash-treated group at day 4 of treatment and remains significantly high compared with baseline value at the end of day 28 (*P* < 0.01). Meanwhile, the OM severity score in the NS oil group revealed a nonsignificant elevation along the study period compared with baseline value and was significantly lower compared with that reported in the control group (*P* < 0.05) at all time points of the treatment period; additionally, the AUC of the NS oil group was significantly lower than that of the control group (5.9 vs. 38.4; *P* < 0.05) (Figure 2(a)). In Figure 2(b), the use of NCI scale revealed that the mean score of OM severity was significantly elevated in the control group at day 4 of treatment compared with the baseline value (*P* < 0.05); meanwhile, the OM severity score was not significantly elevated in the NS oil-treated group at day 4 and was found to be significantly lower than that of the control group. However, both types of treatment produced comparable effects on the OM severity score (based on NCI scale) at the other time points of evaluation (*P* > 0.05). Comparison between the AUC of the two groups showed a significant difference between them along the treatment period (5.44 vs. 8.77; *P* < 0.05) (Figure 2(b)). In other words, the severity of OM was significantly less only at day 4 of treatment in the NS oil group compared with the control group. Based on the evaluation of erythema as an indicator of OM severity, no signs of erythema were reported at baseline in all the studied groups; no significant difference between them was reported at that time (Figure 3(a)). The mean score of erythema was significantly elevated in the magic wash-treated group after day 4 of treatment and remains significantly higher than the baseline value at the end of day 28 (*P* < 0.01). Meanwhile, the severity of erythema in the NS oil group revealed a nonsignificant elevation in the OMAS-E score along the study period compared with the baseline value and was significantly lower compared with that reported in the control group (*P* < 0.05) all over the treatment period; additionally, the AUC of the NS oil group was significantly lower than that of the control group (6.34 vs. 47.9; *P* < 0.001) (Figure 3(a)). Similar pattern of increase in OM severity, when measured in terms of ulceration score using the OMAS-U scale, was observed in both groups during the treatment period; the comparison between the AUC of the two groups also showed a significantly lower value in the NS oil group compared with the control group along the treatment period (6.2 vs. 37.9; *P* < 0.001) (Figure 3(b)). Based on the calculation of total OMAS score (in terms of erythema and ulcer) and the average OMAS (by dividing the total OMAS value/45), Figures 3(c) and 3(d) reveal significantly lower values of OM severity in the NS oil group compared with the control group. Taken together, the severity of OM (calculated in terms of erythema and ulceration) was significantly less all over the treatment period in the NS oil group than that in the control group (Figure 3).  Table 2 showed that the treatment of AML patients with NS oil mouth rinse positively influenced the ability of the patients to consume normal food; this effect was evident in day 4 of treatment, where all the patients (100%) in this group consumed normal food compared with 80% of the patients in the control group (*P*=0.02); this pattern of influence on the food consumption pattern continued all over the study period (Table 2). In Figure 4, the pain score was significantly elevated at day 4 in the control group compared with the baseline value (*P* < 0.05) and declines to comparable values with the baseline after day 12 to the end of treatment. Meanwhile, the pain score in the NS oil-treated group did not show a significant elevation in the VAS pain score compared with the corresponding baseline value all over the treatment period; however, the use of NS oil mouth rinse produces a significant decrease in pain severity on day 12 of treatment upward to the end of the treatment compared with the control group. During the exposure to CT treatment, the AUC of the NS oil-treated group was significantly lower than that reported for the magic mouthwash-treated group (control group) (5.36 vs. 56.2; *P* < 0.001).

### 3.3. Secondary Outcome: Biochemical Scores

**Figure 5:**
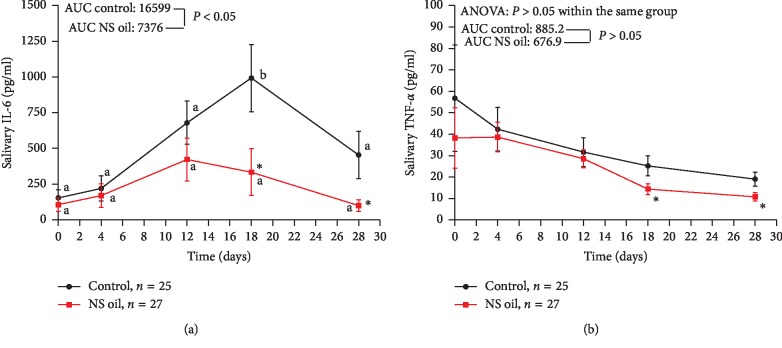
Effect of *Nigella sativa* oil (NS) mouth rinse on (a) salivary IL-6 concentration and (b) salivary TNF-*α* concentration of chemotherapy-induced OM in AML patients. Values are presented as mean ± SD; values with nonidentical letters within the same group are significantly different (*P* < 0.05); ^*∗*^significantly different compared with the control at the same time point (*P* < 0.05).

In Figure 5(a), the salivary IL-6 concentrations of the control were elevated with time during CT treatment, reaching a significantly high level on day 18 compared with baseline value; meanwhile, the elevation of salivary IL-6 in the NS oil-treated group was not significantly different compared with the corresponding baseline value (*P* > 0.05). However, treatment with NS oil mouth rinse significantly reduces salivary IL-6 on day 18 and day 28 compared with the values reported in the control group at the same time points. Additionally, the AUC in the NS oil group was found to be significantly lower than that reported in the control group (7376 vs. 16599; *P* < 0.05).  Figure 5(b) shows that salivary content of TNF-*α* was nonsignificantly decreased in both groups all over the treatment period compared with the baseline values; however, the use of NS oil mouth rinse decreases the salivary TNF-*α* significantly on day 18 and day 28 compared with those reported in the control group at the same time points (*P* < 0.05). Additionally, the AUC in the NS oil group was found to be nonsignificantly different from that reported in the control group (676.9 vs. 885.2; *P* > 0.05).

## 4. Discussion

The clinical outcomes of the present study revealed a significant decrease in the severity of OM in the NS oil-treated group compared with the control group. Although no previous clinical data are available in this regard, similar findings related to the use of other natural compounds for prevention/treatment of CT-induced OM [28, 29] were in tune with the finding of our study. However, it has been noticed in many systematic reviews [9, 30] that different limitations have existed in the previous studies, and despite the obtained positive results in this regard, further well-organized and comprehensive studies are suggested to enable more trustful conclusions. While the principal mechanism of action of NS oil on OM is not well characterized, the effects of its multiple constituents, especially the thymoquinone, demonstrated wound healing [31], analgesic [32], anti-inflammatory [33], and antimicrobial [34] activities may be responsible for its beneficial outcomes. Additionally, different properties such as high antioxidant and antiulceration effects suggested that NS oil be an effective enhancer of the immune functions and a cytoprotectant [35, 36]. Meanwhile, some researchers believed that the combination of these properties with the capacity of NS oil constituents to induce salivation may enable healing and repair of the damaged oral mucosa during cancer chemotherapy [37]. Chemotherapy-induced OM is characterized by inflammatory changes that develop with the direct or indirect effect of the cytotoxic agents and can be associated with severe ulcerations. The pathogenesis of OM is a complex condition which encompasses multiple phases of inflammatory processes that include the vascular, epithelial, ulcerative, and recovery phases [38]. Accordingly, the use of multiple drugs and/or agents with pleiotropic properties, like natural products, is highly suggested in this condition. The present study also showed that using NS oil as mouth rinse significantly and effectively reduces the OM-associated pain compared with the use of magic mouthwash (control group). According to the presented data (Figure 4), no significant difference in pain severity was found between the NS oil-treated and the control groups at baseline; however, a significant difference was observed between the two groups from day 12 until the end of the treatment (day 28). This finding was in tune with those reported by others, which indicated the antinociceptive and analgesic activities of NS oil in various models of nociceptive pain and inflammation [39]. The severity of OM is mostly determined by the type(s) and the dose of the chemotherapeutic agents used. The adopted approach of combining different chemotherapeutic agents enhances the severity of the expected OM [40]. To exclude the possible confounding factors, we enrolled AML patients receiving the same CT protocol, which enables avoiding the differences between different classes of chemotherapeutic agents regarding OM induction [41]. Moreover, the antifungal and antibacterial properties of NS supported its cytoprotective effects in CT-induced OM, and it is both inexpensive and easy to administer, and all participants tolerate it without any significant adverse effects. It has been recognized that maintenance of the swallowing function is critical as an oncologic cure during cancer treatment [42]. In the present study, all the AML patients in the NS oil group were able to maintain the intake of normal food all over the treatment period, which is significantly better than those in the control group. This finding can be correlated with the reduced severity of OM-associated pain and inflammation; additionally, the high oil contents of NS can provide a soothing effect that may be helpful and contribute to better dietary during treatment [43]. Many data from experimental animal models demonstrated the changes in the expression of proinflammatory cytokines such as TNF-*α*, IL-6, and IL-1*β* following the exposure to various CT protocols [44, 45]. However, scanty human data have mentioned the changes of these proinflammatory cytokines in the blood and/or saliva of patients with CT-induced OM [46, 47]. According to a literature review, no previous study has evaluated the changes in the salivary IL-6 and TNF-*α* during the use of NS oil in patients receiving CT for the treatment of AML. The present study revealed that salivary IL-6 concentrations were peaked on day 12 and day 18 in both groups (control and NS oil), which coincided with the progression pattern of OM severity. In both groups, a higher level of IL-6 was also accompanied by a decrease of salivary TNF-*α*; however, the reduction reported in the NS oil group was significantly greater than that in the control group. In this regard, IL-6 seems to play a critical role in the systemic response during tissue injury. However, IL-6 has dual classification as either a proinflammatory or anti-inflammatory cytokine, and it may present the properties of both [48]. Moreover, it has both local and systemic effects on ulcer healing through the stimulation of fibroblast proliferation at the place of injury and is expressed by various cell types in the injured tissues [49]. Considering the small sample size of the present study because of the limited availability of time and AML cases, it is recommended that further studies be conducted with a larger sample size to support the role of topical NS oil in the management of chemotherapy-induced oral mucositis in patients with AML.

## 5. Conclusion

The present study showed for the first time that *Nigella sativa* oil mouth rinse is effective in attenuating the severity of chemotherapy-induced oral mucositis and improves the pain and swallowing function in acute myeloid leukemia patients receiving chemotherapy.

## Figures and Tables

**Table 1:** Demographic characteristic of the participants.

Parameter	Control *n* = 25	NS oil *n* = 27	*P* value
Age (years) (mean ± SD)	37.2 ± 14.4	33.8 ± 13.9	0.38

Age ranges (years), *n* (%)			
<25	5 (20)	9 (33.3)	
25–34	8 (32)	9 (33.3)	
35–44	5 (20)	2 (7.4)	
≥45	7 (28)	7 (26.0)	0.52

Gender, *n* (%)			
Male	11 (44)	16 (59.3)	
Female	14 (56)	11 (40.7)	0.27

BMI, *n* (%)			
Normal	11 (44)	13 (48.1)	
Overweight	6 (24)	5 (18.5)	
Obese	8 (32)	9 (33.3)	0.89

Smoking habit, *n* (%)			
No	18 (72)	21 (77.8)	
Yes	7 (28)	6 (22.2)	0.63

Family history, *n* (%)			
Negative	22 (88)	24 (88.9)	
Positive	3 (12)	3 (11.1)	

Dental status, *n* (%)			
Good	5 (24)	5 (18.5)	
Fair	12 (48)	16 (59.3)	
Bad	7 (28)	6 (22.2)	0.72

Salivary IL-6 (pg/ml) (mean ± SD)	159.8 ± 57.7	106.7 ± 47.2	0.52
Salivary TNF-*α* (pg/ml) (mean ± SD)	56.8 ± 24.8	38.3 ± 14.1	0.51

AML, acute myeloid leukemia; BMI, body mass index; NS, *Nigella sativa*.

**Table 2:** Effects of NS oil mouth rinse on the diet type consumed by the AML patients during chemotherapy.

Diet type	Control	NS oil	Total	*P* value
	*n* (%)	*n* (%)	*n* (%)	
Function—day 0				
Normal	25 (100)	27 (100)	52 (100)	>0.999

Function—day 4				
Normal	20 (80)	27 (100)	47 (90.4)	
Soft	3 (12)	0 (0)	3 (5.8)	
Liquid	2 (8)	0 (0)	2 (3.8)	0.02

Function—day 12				
Normal	15 (60)	27 (100)	42 (80.7)	
Soft	4 (16)	0 (0)	4 (7.7)	
Liquid	6 (24)	0 (0)	6 (11.6)	<0.001

Function—day 18				
Normal	18 (72)	27 (100)	45 (86.5)	
Soft	4 (16)	0 (0)	4 (7.7)	
Liquid	3 (12)	0 (0)	3 (5.8)	0.004

Function—day 28				
Normal	20 (80)	26 (96.3)	46 (88.5)	
Soft	2 (8)	0 (0)	2 (3.8)	
Liquid	3 (12)	1 (3.7)	4 (7.7)	0.159

Notes: values are simple frequency and percentage, and mean ± SD.

## Data Availability

The data utilized to support the findings of the present study are included within the article.

## Abbreviations

AML: Acute myeloid leukemia
AUC: Area under the curve
CT: Chemotherapy
IL-6: Interleukin-6
NS: *Nigella sativa*
OM: Oral mucositis
OMAS: Oral Mucositis Assessment Scale
TNF-*α*: Tumor necrosis factor-alpha.
